# Health outcomes in offspring born to survivors of childhood cancers following assisted reproductive technologies

**DOI:** 10.1007/s11764-020-00929-0

**Published:** 2020-08-26

**Authors:** Greta Sommerhäuser, Anja Borgmann-Staudt, Kathy Astrahantseff, Katja Baust, Gabriele Calaminus, Ralf Dittrich, Marta J. Fernández-González, Heike Hölling, Charlotte J. König, Ralph Schilling, Theresa Schuster, Laura Lotz, Magdalena Balcerek

**Affiliations:** 1 Department of Pediatric Oncology and Hematology, Charité-Universitätsmedizin Berlin, Freie Universität Berlin, Humboldt-Universität zu Berlin, and Berlin Institute of Health, Augustenburger Platz 1, 13353 Berlin, Germany; 2 University Hospital Bonn (UKB), Venusberg-Campus 1, 53127 Bonn, Germany; 3 Department of Obstetrics and Gynecology, Erlangen University Hospital, Friedrich-Alexander University of Erlangen-Nürnberg, Maximiliansplatz 2, 91054 Erlangen, Germany; 4 Robert Koch-Institute Berlin, General-Papestr. 62-66, 12101 Berlin, Germany; 5 Charité-Universitätsmedizin Berlin, Freie Universität Berlin, Humboldt-Universität zu Berlin, and Berlin Institute of Health, Institute for Biometry and Clinical Epidemiology (IBiKE), Charitéplatz 1, 10117 Berlin, Germany; 6 Berlin Institute of Health (BIH), Anna-Louisa-Karsch-Straße 2, 10178 Berlin, Germany

**Keywords:** Assisted reproductive technologies, Childhood cancer survivor, Pediatric cancer, Infertility, Offspring

## Abstract

**Purpose:**

An increasing number of childhood cancer survivors are using assisted reproductive technologies (ART) to overcome treatment-related fertility impairment. We report perinatal and health outcomes of offspring born to survivors following ART.

**Methods:**

The FeCt Multicenter Offspring Study surveyed the health of offspring of childhood cancer survivors. Health outcomes in offspring born to survivors following ART (*n* = 57, 4.6%) or after spontaneous conception (*n* = 1182) were assessed in the German cohort (*n* = 1239) using bivariate analysis. Findings were put into the context of the general German population by health outcome assessment in 1:1 matched-pair analysis (*n* = 2478).

**Results:**

Nearly twice the survivors used ART compared with numbers reported for the German general population (4.6% vs. 2.6%). Successful pregnancies were achieved after a median of two cycles, mainly using non-cryopreserved oocytes/sperm. Multiple sibling births (*p* < 0.001, 28.1% vs. 3.0%) and low birth weight (*p* = 0.008; OR = 2.659, 95% CI = 1.258–5.621) occurred significantly more often in offspring born to survivors who utilized ART than spontaneously conceived children, whereas similar percentages were born preterm or too small for their gestational age. ART did not increase the prevalence of childhood cancer or congenital malformations in offspring born to survivors.

**Conclusion:**

ART use by childhood cancer survivors was successful with both fresh and cryopreserved oocytes/sperm, and did not influence perinatal health or health outcomes when known confounders were taken into account.

**Implications for Cancer Survivors:**

Oncofertility is an important component of patient care. Our study implicates that the utilization of ART by adult survivors of childhood cancer does not put offspring at additional risk for adverse perinatal or health outcomes.

**Electronic supplementary material:**

The online version of this article (10.1007/s11764-020-00929-0) contains supplementary material, which is available to authorized users.

## Introduction

Worldwide, > 5,000,000 live births have been reported following assisted reproductive technologies (ART), mostly in vitro fertilization and intracytoplasmic sperm injection [1], with annual numbers rising [2]. In 2016, 2.6% of children in Germany were born following ART [3]. While the majority of children conceived by ART are born healthy, ART has been associated with a higher risk of adverse birth outcomes than spontaneous conception [4]. These include increased occurrences of adverse perinatal events [5], childhood cancer [6], congenital malformations [7] (including heart defects [8]), hypertension [9, 10], and asthma [11]. Hypotheses explaining these observations implicate pharmaceutically induced ovulation, micromanipulation during ART procedures, effects associated with increased incidence of multiple sibling births [4], and subfertility itself [12].

Infertility is a known late effect of cancer treatment in childhood [13–15], yet national [16, 17] and international [18, 19] guidelines on fertility protection have only been developed in recent years. An increasing number of childhood cancer survivors are employing ART to fulfill the wish for biological children [20]. Survivors report being anxious about the general health of future children and the possibility that their previous cancer treatment may increase the risk of cancer in offspring [21]. Studies have shown that offspring of non-hereditary childhood cancer survivors do not have an increased risk for malformations, genetic diseases, or non-hereditary cancers [22–24]. Yet, whether ART affects offspring health when used by childhood cancer survivors has not been specifically addressed.

Within our *FeCt*^1^*Multicenter Offspring Study*, we compared perinatal outcomes and the prevalence of childhood cancer and congenital malformations, including heart defects, in children born to German survivors following ART and after spontaneous conception. To contextualize our findings, we also compared health outcomes in the survivor offspring cohort to children in the general German population.

## Methods

### Design and participants

Between 2013 and 2019, we conducted the FeCt Multicenter Offspring Study in Germany, Austria, Czech Republic, Poland, and Switzerland to survey childhood cancer survivors on health aspects of their biological children. The study was approved by the *Charité* local ethics committee (EA2/237/05, EA2/103/11) and the German Society for Pediatric Oncology and Hematology. Survey methods of the German cohort and our study questionnaire were published previously [21, 25, 26]. Childhood cancer survivors identified as having biological children in the previously conducted nationwide studies, FeCt on survivor fertility [21, 26] and *VIVE* on somatic and psycho-social late effects in survivors [27], were contacted via the German Childhood Cancer Registry (*n* = 1340, Supplemental Figure 1). Survivors of at least 18 years of age were included for study participation if informed consent was given. Participating survivors who had used ART were additionally interviewed by phone in June and July 2019 on details of their ART treatment and course of pregnancy. For comparison with the general German population, we drew data from the KiGGS Study (health examination survey for children and adolescents in Germany) conducted in 17,641 children by the *Robert Koch Institute* between 2003 and 2006 [28]. Survivor offspring (*n* = 1239) were matched 1:1 with children from the KiGGS collective using gender, age, and born as singletons versus as multiple siblings in a case-control design for matched-pair analysis.

### Variables

In total, 46 items concerning diseases, pain, well-being, living conditions, diet, health-related behavior, healthcare utilization, and social determinants were measured. Core data for the participating survivors were available from the German Childhood Cancer Registry. Participating survivors reported whether children were conceived spontaneously or via ART (specifying type). World Health Organization definitions were used to describe perinatal outcomes (gestational age, birth weight, small for gestational age) [29] and to categorize congenital malformations including heart defects (International Classification of Disease, *ICD-10*) [29]. The International Classification of Childhood Cancer (ICCC-3) was used to specify cancer diagnoses [30]. Whether survivors’ children had a cancer, malformation, or heart condition were surveyed by yes/no questions, and the diagnosis was further described in answer to “If yes, which?”. Whether survivors (who used ART) received chemotherapy, radiotherapy, and/or stem cell transplantation to treat their cancers were available via cooperation with the completed FeCt and VIVE studies (Supplemental Figure 1). Cause and type of infertility and details about ART procedure and pregnancy/birth-related complications were queried via phone interview.

### Statistical methods

Analyses were performed using IBM SPSS Statistics, Version 25. Questionnaires lacking child’s gender, age or mode of conception, or (for comparability with the *KiGGS* cohort, age range 0–17 years) describing children ≥ 18 years at survey time were excluded from analyses (Supplemental Figure 1). To minimize the amount of missing data, a user-friendly questionnaire containing standardized and validated instruments from the *KiGGS Study* was designed and a pilot study was performed. Cases with missing data were omitted and remaining data were analyzed (listwise deletion). Survivor offspring group differences were nonparametrically tested (2-sided chi-square and Mann-Whitney *U* tests for unpaired samples) with a 5% significance level. Even though the number of offspring born to childhood cancer survivors following ART (*n* = 57) was small, post hoc calculation of statistical power revealed that the given sample size provided 80% power to detect group differences at the 5% significance level and an odds ratio (OR) of 2.8 (medium effect) by logistic regression. Bivariate analyses were carried out using McNemar and Wilcoxon signed-rank tests for paired nonparametric samples with a significance level of < 5% to detect group differences. Effect sizes were calculated including unadjusted OR with 95% confidence intervals (95% CI) and Cohen’s *d* effect size [31] (thresholds: 0.2 = small, 0.5 = medium, 0.8 = large). Binary logistic regression was carried out to estimate adjusted ORs with 95% CI in multivariate analyses to assess the intervariable dependencies of the sociodemographic factors, gender, age, migration background, and educational attainment of parents (rated low, medium, or high using CASMIN classification [32], as well as multiple sibling birth, smoking/drinking habits during pregnancy, and parental estimation of their child’s health). The age at diagnosis (grouped into 0–4, 5–9 and 10 or more years of age) and type of cancer (grouped into leukemia/lymphoma, brain tumors and extra-cranial solid tumors) in the parents were considered additional variables in multivariate analysis.

## Results

### Participants

We received responses from 852 German childhood cancer survivors (65.8% of those contacted), who returned 1340 questionnaires for their children (Supplemental Figure 1). Responders were more likely to be female (*p* < 0.001) and moderately to highly educated (*p* < 0.001). Survivors’ diagnoses, age at diagnosis, and at time of survey were equally distributed among survey participants and non-participants (Supplemental Table 1). Of the 1239 children born to childhood cancer survivors, 57 were conceived after ART (4.6%) and were born, on average, closer to the time of survey (Table 1). Compared with those who spontaneously conceived, survivors using ART experienced more multiple sibling births, and none reported smoking or drinking during pregnancy. Survivors who utilized ART were older at cancer diagnosis, but parental cancer diagnoses were equally distributed between children born after ART or spontaneously conceived children (Table 1). Of 40 survivors who reported ART, we successfully interviewed 27 survivors (67.5%, Supplemental Figure 1) about their 44 children born after ART. Clinical records available for 24 interviewed survivors showed that all received chemotherapy, 12 had also received radiotherapy and one underwent bone marrow transplantation. In both female and male childhood cancer survivors, male factor infertility contributed more strongly to the necessity for ART in couples including a childhood cancer survivor (Table 2). The majority of survivors used fresh oocytes/sperm for fertility treatment. In vitro fertilization/intracytoplasmic sperm injection was successful after one cycle in half the couples; however, 18.2% (4/22 pregnancies in female survivors) required ≥ 3 cycles. Most childhood cancer survivors that used ART reported spontaneous vaginal deliveries (65.4% in female, 66.7% in male survivors), with only two emergency cesarean sections being reported (Table 2). Paired analysis of survivor offspring matched to children in the general German population (KiGGS cohort) showed a higher educational level in parents who survived childhood cancer than in the general population (Table 1). Survivors were also more careful about alcohol and tobacco use during pregnancy (Table 1).

**Table 1:** Characteristics describing the offspring analyzed

	**Childhood cancer survivor offspring**				*p*	**Paired analysis (matched 1:1)**				*p*
	Conceived spontaneously		Conceived by ART			Children from the *KiGGS* collective		Childhood cancer survivor offspring		
	Missing data (*n*)	*n* (%)	Missing data (*n*)	*n* (%)		Missing data (*n*)	*n* (%)	Missing data (*n*)	*n* (%)	
**Total**		1,182 (100.0)		57 (100.0)			1,239 (100.0)		1,239 (100.0)	
**Gender**^a^	-		-		0.686	-		-		1.0
Female		569 (48.1)		29 (50.9)			598 (48.3)		598 (48.3)	
Male		613 (51.9)		28 (49.1)			641 (51.7)		641 (51.7)	
**Year of birth**					< 0.001	-		-		< 0.001
1985 to 1999		59 (5.0)		1 (1.8)			747 (60.3)		60 (4.8)	
2000 to 2009		561 (47.5)		12 (21.1)			492 (39.7)		573 (46.2)	
≥ 2010		562 (47.5)		44 (77.2)			-		606 (48.9)	
**Age at time of survey**^a^	-		-		< 0.001	-		-		1.0
Mean age (SD)		5.8 [4.1]		3.9 [3.4]			5.7 (4.13)		5.7 (4.13)	
Median age (IQR)		5 [5]		3 [3]			5 (6)		5 (6)	
0 to 6		771 (65.2)		48 (84.2)			819 (66.1)		819 (66.1)	
7 to 13		330 (27.9)		8 (14.0)			338 (27.3)		338 (27.3)	
≥ 14		81 (6.9)		1 (1.8)			82 (6.6)		82 (6.6)	
**Multiple sibling birth**^a^	-	35 (3.0)	-	16 (28.1)	< 0.001	-	51 (4.1)	-	51 (4.1)	1.0
**Migration background**	1	240 (20.3)	-	10 (17.5)	0.610	1	267 (21.6)	1	250 (20.2)	0.439
**Educational attainment**	-		-		0.199	12		-		0.001
Low		170 (14.4)		4 (7.0)			162 (13.2)		174 (14.0)	
Medium		520 (44.0)		26 (45.6)			679 (55.3)		546 (44.1)	
High		492 (41.6)		27 (47.4)			386 (31.5)		519 41.9	
**Smoking during pregnancy**	12	90 (7.7)	1	-	0.031	22	203 (16.7)	13	90 (7.3)	< 0.001
**Drinking during pregnancy**	11	43 (3.7)	1	-	0.144	17	198 (16.2)	12	43 (3.5)	< 0.001
**Year of diagnosis (survivor parent)**	1		-		0.796	-	-	1		-
1980 to 1989		694 (58.8)		34 (59.6)					728 (58.8)	
1990 to 1999		462 (39.1)		23 (40.4)					485 (39.1)	
≥ 2000		25 (2.1)		-					25 (2.0)	
**Age at diagnosis (survivor parent)**	1		-		0.032	-	-	1		-
Mean age (SD)		8.5 [4.5]		9.7 [4.3]					8.6 (4.5)	
Median age (IQR)		9 [7]		10 [6]					9 (8)	
0 to 4		284 (24.0)		12 (21.1)					296 (23.9)	
5 to 9		325 (27.5)		8 (14.0)					333 (26.9)	
≥ 10		572 (48.4)		37 (64.9)					609 (49.2)	
**Diagnosis (ICCC-3)**	1		-		0.541	-	-	1		-
Leukemia		486 (41.2)		29 (50.9)					515 (41.6)	
Lymphomas		182 (15.4)		8 (14.0)					190 (15.3)	
Brain tumors		86 (7.3)		5 (8.8)					91 (7.3)	
Neuroblastoma		46 (3.9)		1 (1.8)					47 (3.8)	
Retinoblastoma		23 (1.9)		-					23 (1.9)	
Renal tumors		70 (5.9)		4 (7.0)					74 (6.9)	
Hepatic tumors		3 (0.3)		-					3 (0.2)	
Bone tumors		112 (9.5)		6 (10.5)					118 (9.5)	
Soft tissue tumors		114 (9.7)		1 (1.8)					115 (9.3)	
Germ cell tumors		38 (3.2)		3 (5.3)					41 (3.3)	
Other malignant epithelial neoplasm		21 (1.8)		-					21 (1.7)	
Other neoplasm (unspecified)		-		-					-	

*ART*, assisted reproductive technologies; *SD*, standard deviation; *IQR*, interquartile range; *ICCC-3*, International Classification of Childhood Cancer, third revision (https://seer.cancer.gov/iccc/)

^a^Matching variables for paired analysis

**Table 2:** ART characteristics (telephone interview)

**Characteristics**	**Offspring born to survivors using ART**				
	Female survivor (parent)		Male survivor (parent)		
	Missing data (*n*)	*n* (%)	Missing data (*n*)	*n* (%)	*p*
**Total of offspring born after ART**^**a**^		26 (100.0)		18 (100.0)	
**Gender of offspring**	-		-		0.179
Female		12 (46.2)		12 (66.7)	
Male		14 (53.8)		6 (33.3)	
**Infertility diagnosed in survivor**	1	10 (40.0)	-	14 (77.8)	0.014
Female factor		7 (28.0)		2 (22.2)	
Male factor		15 (60.0)		13 (72.2)	
Both female and male factor		3 (12.0)		1 (5.6)	
**ART**	-		-		-
IVF		3 (11.5)		4 (22.2)	
ICSI		17 (65.4)		10 (55.5)	
ICSI and TESE^a^		3 (11.5)		-	
ICSI (with donor sperm)		-		1 (5.5)	
IUI		3 (11.5)		1 (5.5)	
IUI (with donor sperm)		-		2 (11.1)	
**Use of sperm or oocytes**	-		-		0.025
Fresh		23 (88.5)		10 (55.6)	
Cryopreserved before cancer treatment		-		3 (16.7)	
Cryopreserved after cancer treatment		3 (11.5)		5 (27.8)	
**Number of IVF/ICSI treatment cycles**	1		-		0.187
1		10 (45.5)		8 (50.0)	
2 to 3		8 (36.4)		8 (50.0)	
≥ 4		4 (18.2)		-	
**Pregnancy complications**	-	8 (30.8)	-	-	0.009
Gestational diabetes (non-insulin dependent)		3 (11.5)		-	
Gestational diabetes (insulin dependent)		3 (11.5)		-	
Premature contractions		1 (3.8)		-	
Vanishing twin syndrome		1 (3.8)		-	
**Birth mode**	-		-		-
Vaginal delivery		17 (65.4)		12 (66.7)	
Vacuum extraction		1 (3.8)		1 (5.5)	
Elective cesarean section		3 (11.5)		4 (22.2)	
Cesarean section with medical indication		4 (15.4)		-	
Emergency cesarean section		1 (3.8)		1 (5.5)	
**Year of birth (survivor parent)**					0.140
1960 to 1969		2 (7.7)		3 (16.7)	
1970 to 1979		9 (34.6)		10 (55.6)	
≥ 1980		15 (57.7)		5 (27.8)	
**Age at diagnosis (survivor parent)**	-				0.434
Mean age (SDF)		9.0 [4.5]		10.2 [4.3]	
Median age (IQR)		9.5 [9.0]		12.5 [6.5]	
**Diagnosis (grouped)**	-				0.565
Leukemia/lymphomas		18 (69.2)		15 (83.3)	
Brain tumors		3 (11.5)		1 (5.6)	
Solid tumors		5 (19.2)		2 (11.1)	
**Treatment including radiotherapy**	3	11 (47.8)	1	7 (41.2)	0.676
Chemotherapy only		12 (52.2)		10 (58.8)	
Chemo- and radiotherapy		11 (47.8)		5 (29.4)	
Chemotherapy, radiotherapy and BMT		-		2 (11.8)	-
**Age at birth of offspring (in total)**	-		-		0.001
Mean age (SDF)		31.1 [2.5		35.0 [3.7]	
Median age (IQR)		31 [3.3		34 [6.5]	
Partner’s age at birth of offspring (in total)	-		-		0.032
Mean age (SDF)		35.6 [4.9]		32.6 [2.3]	
Median age (IQR)		36 [9.25]		32 [3.0]	

*ART*, assisted reproductive technologies; *IVF*, in vitro fertilization; *ICSI*, intracytoplasmic sperm injection; *TESE*, testicular sperm extraction; *IUI*, intrauterine insemination; *BMT*, bone marrow transplant; *SD*, standard deviation; *IQR*, interquartile range

### Perinatal outcomes

We evaluated the prevalence of preterm birth, low birth weight, and small for gestational age in survivor offspring from the survey responses. Children born to survivors, whether following ART or spontaneous conception, were born to term (37 to < 42 weeks of gestation, Table 3). The offspring born following ART, however, were delivered slightly but significantly earlier (mean 38.5 vs. 39.1 weeks of gestation, *p* = 0.028; Cohen’s *d* 0.127). While using ART did not significantly increase the risk for premature birth among childhood cancer survivors, bivariate analysis revealed that birth weight below 2500 g (low birth weight) was statistically more prevalent (*p* = 0.008; OR = 2.659, 95% CI = 1.258–5.621, Table 3). In multivariate analyses, however, differences in the prevalence of low birth weight between groups were no longer apparent (Supplementary Table 2). Preterm birth (*p* < 0.001; OR = 38.306, 95% CI = 21.044–69.727) and congenital heart defects (*p* = 0.046; OR = 5.616, 95% CI = 1.030–30.610) were confirmed to be confounding variables affecting the prevalence of low birth weight in our cohort. When birth weight was related to the respective gestational age to identify children born too small for gestational age (< 10th percentile), no differences were detected between survivor offspring born after ART or spontaneous conception (Table 3). Paired analysis with the general population (*KiGGS* children) revealed that survivor offspring were delivered at term, although marginally earlier (mean 39.0 vs. 39.3 weeks of gestation, *p* = 0.036; Cohen’s *d* = 0.065, Table 3). Among survivor offspring, the risk of prematurity (< 37 weeks of gestation, *p* = 0.008; OR = 1.701, 95% CI = 1.253–2.308) and low birth weight (< 2500 g, *p* = 0.047; OR = 1.431, 95% CI = 1.020–2.007) was slightly but significantly elevated compared with the general population. Multivariate analyses confirmed group differences for prematurity (*p* < 0.001; OR = 2.002, 95% CI = 1.395–2.874), but not for low birth weight which was instead shown to be influenced by the epidemiologically known confounders, prematurity, and multiple sibling births [33] (Supplementary Table 2). The prevalence of being born too small for gestational age did not differ between survivor offspring and children in the general population.

**Table 3:** Perinatal outcomes in offspring (bivariate analyses)

**Objectives**	**Childhood cancer survivor offspring**				Effect size (95% CI)	*p*	**Paired analysis (matched 1:1)**				Effect size (95% CI)	*p*
	Conceived spontaneously		Conceived by ART				Children from the *KiGGS* collective		Childhood cancer survivor offspring			
	Missing data (*n*)	*n* (%)	Missing data (*n*)	*n*			Missing data (*n*)	*n* (%)	Missing data (*n*)	*n* (%)		
**Total**		1,182 (100.0)		57 (100.0)				1,239 (100.0)		1,239 (100.0)		
**Gestational age**	39		1		Cohen’s *d* 0.127	0.028	137		40		Cohen’s *d* 0.065	0.036
Mean gestational age (SD)		39.1 [2.2]		38.5 [2.2]				39.3 [1.9]		39 [2.2]		
Median gestational age (IQR)		40 [2]		39 [2]				40 [1]		39 [2]		
Preterm birth (< 37 weeks gestation)^a^		115 (10.1)		9 (16.1)		0.149		70 (6.4)		124 (10.3)	OR 1.701 (1.253–2.308)	0.008
Extremely preterm (< 28 weeks gestation)		6 (0.5)		-				6 (0.5)		6 (0.5)		
Very preterm (28 to < 32 weeks gestation)		10 (0.9)		-				4 (0.4)		10 (0.8)		
Moderate preterm (32 to < 37 weeks gestation)		99 (8.7)		9 (16.1)				60 (5.4)		108 (9.0)		
Term (37 to < 42 weeks gestation)		958 (83.8)		45 (80.4)				971 (88.1)		1003 (83.7)		
Post term (42 weeks gestation or more)		70 (6.1)		2 (3.6)				61 (5.5)		72 (6.0)		
**Birth weight**	13		-			0.075	21		13			0.069
Mean birth weight in grams (SD)		3,340 [582.9]		3,90 [599.3]				3,410 [576.5]		3,334 [584.3]		
Median birth weight in grams (IQR)		3350 [685]		3310 [775]				3420 [670]		3350 [691]		
**Low birth weight (< 2500 g)**^a^		77 (6.6)		9 (15.8)	OR 2.659 (1.258–5.621)	0.008		61 (5)		86 (7)	OR 1.431 (1.020–2.007)	0.047
Extremely low birth weight (< 1000 g)		5 (0.4)		-				5 (0.4)		5 (0.4)		
Very low birth weight (1000 to < 1500 g)		7 (0.6)		-				5 (0.4)		7 (0.6)		
Moderately low birth weight (1500 to < 2500 g)		65 (5.6)		9 (15.8)				51 (4.2)		74 (6.0)		
Normal birth weight (2500 to < 4000 g)		957 (81.9)		43 (75.4)				983 (80.7)		1000 (81.6)		
High birth weight (4000 g or more)		135 (11.5)		5 (8.8)				174 (14.3)		140 (11.4)		
Small for gestational age^b^	48	81 (7.1)	1	7 (12.5)		0.135	139	74 (6.7)	49	88 (7.4)	-	0.616

*ART*, assisted reproductive technologies; *CI*, confidence interval; *SD*, standard deviation; *IQR*, interquartile range; *OR*, odds ratio

^a^World Health Organization definitions were employed (https://www.who.int)

^b^Small for gestational age was defined as a birth weight below the 10th percentile for the average at the respective gestational age

### Prevalence of childhood cancer

All childhood cancer survivors surveyed submitted information about cancer occurrences in their children. Although eight children born to survivors (0.6%) were diagnosed with cancer before the age of 18 (including two retinoblastomas in children with hereditary predispositions), none of the affected children were born following ART (Table 4). Among the children from the *KiGGS* cohort used in paired analysis, one child was diagnosed with a brain tumor and a second child with renal tumor (0.2%, *p* = 0.180). This comparison indicates that childhood cancer prevalence is no different in survivor offspring than in the general German population.

**Table 4:** Health outcomes in offspring (bivariate analyses)

**Objectives**	**Childhood cancer survivor offspring**				Effect size (95% CI)	*p*	**Paired analysis (matched 1:1)**				Effect size (95% CI)	*p*
	Conceived spontaneously		Conceived by ART				Children from the *KiGGS* collective		Childhood cancer survivor offspring			
	Missing data (*n*)	*n* (%)	Missing data (*n*)	*n* (%)			Missing data (*n*)	*n* (%)	Missing data (*n*)	*n* (%)		
**Total**		1,182 (100.0)		57 (100.0)				1,239 (100.0)		1,239 (100.0)		
**Childhood cancer** (ICCC-3 *2008*)	-	8 (0.7)		-		0.533	168	2 (0.2)	-	8 (0.6)		0.180
**Non-hereditary childhood cancer**^a^	1	5 (0.4)	-	-		0.623	-	2 (0.2)	1	5 (0.4)		0.687
**Diagnosis of childhood cancer**							-		1			
Leukemia		-						-		-		
Lymphomas		1^d^						-		1^d^		
Brain tumors		1^d^						1^d^		1^d^		
Neuroblastoma		1^d^						-		1^d^		
Retinoblastoma^a^		2^d^						-		2^d^		
Renal tumors		1^d^						1^d^		1^d^		
Hepatic tumors		-						-		-		
Bone tumors		-						-		-		
Soft tissue tumors		1^d^						-		1^d^		
Germ cell tumors		-						-		-		
Other malignant epithelial neoplasm		-						-		-		
Other neoplasm (unspecified)		-						-		-		
**Congenital malformations** (Q00-Q99, ICD-10 2016)	15	69 (5.9)	-	2 (3.5)		0.448	168	119 (11.1)	15	71 (5.8)	OR 0.393 (0.284 to 0.544)	< 0.001
**Diagnosis**	4		-				-		4			
Nervous system		1 (1.4)		1^d^				2 (1.5)		2 (2.7)		
Eye, ear, face, and neck		3 (4.2)		-				7 (5.4)		3 (4.0)		
Circulatory system^b^		28 (38.9)		1^d^				38 (29.2)		29 (38.7)		
Respiratory system		-		-				-		-		
Cleft lip and cleft palate		4 (5.6)		-				3 (2.3)		4 (5.3)		
Digestive system		3 (4.2)		1^d^				3 (2.3)		4 (5.3)		
Genital organs		5 (6.9)		-				11 (8.5)		5 (6.7)		
Urinary system		6 (8.3)		-				11 (8.5)		6 (8.0)		
Musculoskeletal system		20 (27.8)		-				48 (36.9)		20 (26.7)		
Other congenital malformations		1 (1.4)		-				6 (4.6)		1 (1.3)		
Chromosomal abnormalities (not elsewhere classified)		1 (1.4)		-				1 (0.8)		1 (1.3)		
Number of congenital malformations reported^c^		72 (6.2)		3 (5.3)				130 (12.1)		75 (6.1)		
Number of children with congenital malformations		65 (5.6)		2 (3.5)				119 (11.1)		67 (5.5)		
**Congenital heart defects** (Q20-28, ICD-10 2016)	15	23 (2.0)	-	1^d^		0.908	168	35 (3.3)	15	24 (2.0)	OR 0.592 (0.350 to 1.002)	0.049
**Diagnosis**	-		-				-		-			
Cardiac chambers and connections		-		-				1 (2.6)		-		
Cardiac septa		13 (46.4)		1^d^				28 (73.7)		14 (48.3)		
Pulmonary, tricuspid valves, aortic and mitral valves		5 (17.9)		-				2 (5.3)		5 (17.2)		
Other congenital malformations of heart		1 (3.6)		-				1 (2.6)		1 (3.4)		
Great arteries and great veins		9 (32.1)		-				5 (13.2)		9 (31.0)		
Other malformations of peripheral vascular system		-		-				1 (2.6)		-		
Other malformations of circulatory system		-		-				-		-		
Number of reported congenital heart defects^c^		28		1^d^				38 (3.5)		29 (2.4)		
Numbers of children with congenital heart defects		23 (2.0)		1^d^				35 (3.3)		24 (2.0)		

*ART*, assisted reproductive technologies; *CI*, confidence interval; *ICCC-3*, International Classification of Childhood Cancer, third revision (https://seer.cancer.gov/iccc/); *SD*, standard deviation; *IQR*, interquartile range; *ICD-10*, International Statistical Classification of Diseases, 10th revision; *OR*, odds ratio

^a^Two diagnoses (retinoblastomas) with hereditary predispositions were excluded

^b^Diagnoses of congenital malformations of the circulatory system are reported in detail in congential heart defects

^c^Children with multiple diagnoses appear more than once in the table

^d^Numbers (*n*) were too small to report meaningful percentages

### Prevalence of congenital malformations and heart defects

The childhood cancer survivors surveyed also submitted information about the occurrence of congenital malformations and heart defects in their offspring. Neither congenital malformations nor heart defects were more prevalent in survivor offspring born following ART compared with those spontaneously conceived (Table 4). The surveyed survivors reported 75 diagnoses of congenital malformations in 71 offspring of the 1224 offspring informed on in the survey (5.8%). In the paired analysis for children from the general German population (*KiGGS* cohort), 130 diagnoses of congenital malformations were reported in 119 children, yielding an even higher prevalence for congenital malformations (11.1%, *p* < 0.001; OR = 0.393, 95% CI = 0.284–0.544, Table 4). The slightly higher prevalence for congenital malformations in the *KiGGS* cohort remained significant (*p* = 0.002, OR = 0.538, 95% CI = 0.364–0.796) in multivariate analyses (Supplementary Table 3). Congenital malformations were shown to be associated with a lower parental estimation of the child’s overall health (“very good” *p* < 0.001; OR = 0.312, 95% CI = 0.161–0.604). Significantly more congenital heart defects (bivariate analysis: *p* = 0.049; OR = 0.592, 95% CI = 0.350–1.002) occurred in the general population, with 38 diagnoses in 35 *KiGGS* children reported (total = 1071, 3.3%) in comparison with 29 diagnoses in 24 survivor offspring (total = 1224, 2.0%, Table 4). Multivariate analysis, however, did not confirm this higher prevalence for congenital heart defects in *KiGGS* children (Supplementary Table 3). The prevalence of congenital malformations or heart defects was not increased in offspring of childhood cancer survivors in the surveyed cohort in any group comparisons conducted.

## Discussion

Here, we specifically investigate the impact of ART on the health of offspring born to childhood cancer survivors using the only survey information published to date that was tailored to this question. Nearly twice the childhood cancer survivors used ART in our surveyed cohort (4.6%) compared with numbers reported by the German In Vitro Fertilization Registry (2.6%) [34]. This distribution parallels findings from Melin et al., who compared fertility treatments among female survivors of adult cancer and their siblings (5.2% vs. 2.8%) [35]. While current guidelines recommend fertility preservation before cancer treatment [36], patients in our cohort were treated (1980–1999) before its routine implementation. Only few patients cryopreserved oocytes/sperm prior to cancer treatment. In line with results from a recent European study [37], more male survivors cryopreserved, likely because sperm collection is less invasive and costly, but possibly also due to differences in counseling [38]. Our data showed that even in cases lacking pretreatment oocytes/sperm cryopreservation, successful ART pregnancies occurred in childhood cancer survivors. The increasing numbers of childhood cancer survivors who turn to ART [34] stress the importance of establishing an information base from which to counsel childhood cancer patients and survivors.

Our analyses revealed that the prevalence of multiple sibling births after ART was nearly tenfold higher than for children conceived spontaneously (28.1% vs. 3%). The higher prevalence of multiple sibling births after ART parallels the 34% reported for the general population in 2016 by the German In Vitro Fertilization Registry [34] and is in line with the 31.5% prevalence reported in 2016 in the USA [39]. Recent studies describe a mediating effect of multiple sibling births after ART on adverse obstetric outcomes [4, 5], indicating relevance for health also reflected in our findings from multivariate analyses on perinatal outcomes (*p* < 0.001; OR = 3.902, 95% CI = 1.804 to 8.441, Supplementary Table 1). When known confounders including multiple sibling birth were taken into account [4, 5, 40–43], perinatal outcomes were no different in survivor offspring, whether conceived after ART or spontaneously. We detected a modest increase in the prevalence for moderate preterm birth (32 to < 37 weeks of gestation) in childhood cancer survivors compared to the *KiGGS* cohort, as a representative for the general German population. These findings are reassuring, since most medical consequences occur in very (28 to < 32 weeks of gestation) or extremely (< 28 weeks of gestation) preterm infants [44]. Recent studies have cited long-term treatment effects as a possible source for their detected increased prevalence of adverse pregnancy outcomes in childhood cancer survivors. Van de Loo et al. and van Dorp et al. both found that childhood cancer survivors exposed to radiation were at higher risk for preterm delivery and low birth weight [24, 45]. The majority of survivors in our collective (58.7%) were treated in the 1980s, typically receiving higher doses of radiation and alkylating agents (in multiagent chemotherapy schedules) than are currently used [46]. Against this backdrop of progressively reduced toxicity regimens, our findings appear particularly reassuring for patients treated with the less toxic protocols for childhood cancer today. Our study confirms the known increase in multiple sibling births following ART and indicates that prevalence was no higher in childhood cancer survivors than in the general German population. Adverse obstetric outcomes in survivors, as described by preceding studies, were only reflected by a small increase of moderate preterm births in our survivor cohort.

We found that ART did not raise the prevalence of childhood cancer among survivor offspring. In this respect, currently available data collected for the general population are not consistent. Williams et al. reported no increase in the overall risk for cancer among 106,013 children conceived through ART in the British general population, while the risk for hepatoblastoma and rhabdomyosarcoma appeared slightly increased [47]. Likewise, Spaan et al. reported no increase in the overall cancer risk among 24,269 children conceived after ART in the general Dutch population after a median follow-up of 21 years [48]. A meta-analysis of 25 cohort and case-control studies published in 2013, however, calculated a slightly elevated overall risk for cancer in children born after ART (RR = 1.33, 95%CI = 1.08–1.63) [49]. The authors pointed out that subfertility and potential epigenetic defects in the gametes, rather than the ART procedure itself, might be the most important predisposing factor for childhood cancer underlying these data [49]. While a meta-analysis by Hoorsan et al. from 2017 arrived at a 53% higher risk for congenital malformations following ART in the general population [50], our data showed no increased risk for congenital malformations or heart defects among offspring born to childhood cancer survivors who used ART. Hoorsan et al. discuss genetic characteristics and conditions specifically occurring in infertile couples as causative, which may differ from characteristics of our study population. When comparing the prevalence of childhood cancer and congenital malformations in survivor offspring with children from the KiGGS Study, whether conceived by ART or spontaneous conception, our study confirmed findings from large studies [22–24], that showed no higher prevalence for childhood cancer in survivor offspring than in children in the general population. Similarly, the prevalence of congenital malformations, including heart defects, was not elevated in offspring born to our survivor cohort than the reported prevalence in the general population, in line with currently available data [51–55]. Our findings in the survivor cohort and in comparisons with the general German population support that the use of ART by childhood cancer survivors does not put offspring at additional risk for adverse health outcomes including childhood cancer or congenital malformations.

The study setting and conduct feasibility among childhood cancer survivors in Germany posed certain limitations. Recruitment was based on previous surveys identifying survivors with biological children, potentially causing a selection bias. This approach was necessary to reduce the study burden for survivors, as required by the German Society for Pediatric Oncology and Hematology. The questionnaire-based setting could produce recall bias that could reduce data accuracy. However, all survivor parents had been treated according to standardized trial protocols, for which detailed treatment information was available through the German Society for Pediatric Oncology and Hematology. Although we were able to examine a number of potential mediating factors, we had no information on maternal age, body mass index, and infections during pregnancy, which are further factors influencing perinatal events [46, 56–58]. Missing data of the main outcomes were rare in survivor offspring: 1.79% (0.08–3.95%) but more prevalent in the KiGGS cohort: 10.78% (1.70%–13.56%). Although we have no indication of this, it cannot be ruled out with certainty that these were not completely at random. Despite the explorative character of this study, which does not allow for confirmatory conclusions, our analyses offer new insights into health issues in offspring born to childhood cancer survivors, and the high response rate reflects the strong interest shown by survivors in these issues. The small sample size of 57 survivor offspring born after ART was adequate to detect medium effects (*d* 0.5 or higher). Future studies are needed to further explore the effect of ART within a larger population. Our study delivers encouraging results for survivors of childhood cancer that demonstrate that the vast majority of offspring born to survivors do not experience adverse perinatal outcomes or later health problems, independently of whether conception was spontaneous or required ART.

## Electronic supplementary material

ESM 1 (PDF 222 kb)

## Data Availability

The datasets generated during and/or analyzed during the current study are available from the corresponding author on reasonable request.

## Footnotes

1 FeCt, fertility after chemo- and radiation therapy in childhood and adolescence
