# Supplementary material for: Health outcomes in offspring born to survivors of childhood cancers following assisted reproductive technologies
Source: J Cancer Surviv. 2020 Aug 26;15(2):259–72. doi: 10.1007/s11764-020-00929-0 (PMC7966626; doi:10.1007/s11764-020-00929-0)
Supplement: Supplementary file 1 — (PDF 222 kb) [file 11764_2020_929_MOESM1_ESM.pdf]

Journal of Cancer Survivorship

Health outcomes in offspring born to survivors of childhood cancers following assisted reproductive technologies

Greta Sommerhäuser; Anja Borgmann-Staudt; Kathy Astrahantseff; Katja Baust; Gabriele Calaminus; Ralf Dittrich; Marta J. Fernández-González; Heike Hölling; Charlotte J. König; Ralph Schilling; Theresa Schuster; Laura Lotz; Magdalena Balcerek

Corresponding author Prof. Dr. med. Anja Borgmann-Staudt

Charité-Universitätsmedizin Berlin

Department of Pediatric Oncology and Hematology

Augustenburger Platz 1, 13353 Berlin

Telephone: +49 30 450 666 204 / Email: anja.borgmann@charite.de

Supplemental Figure 1. CONSORT diagram on cohort enrolment, response behavior and exclusion criteria.

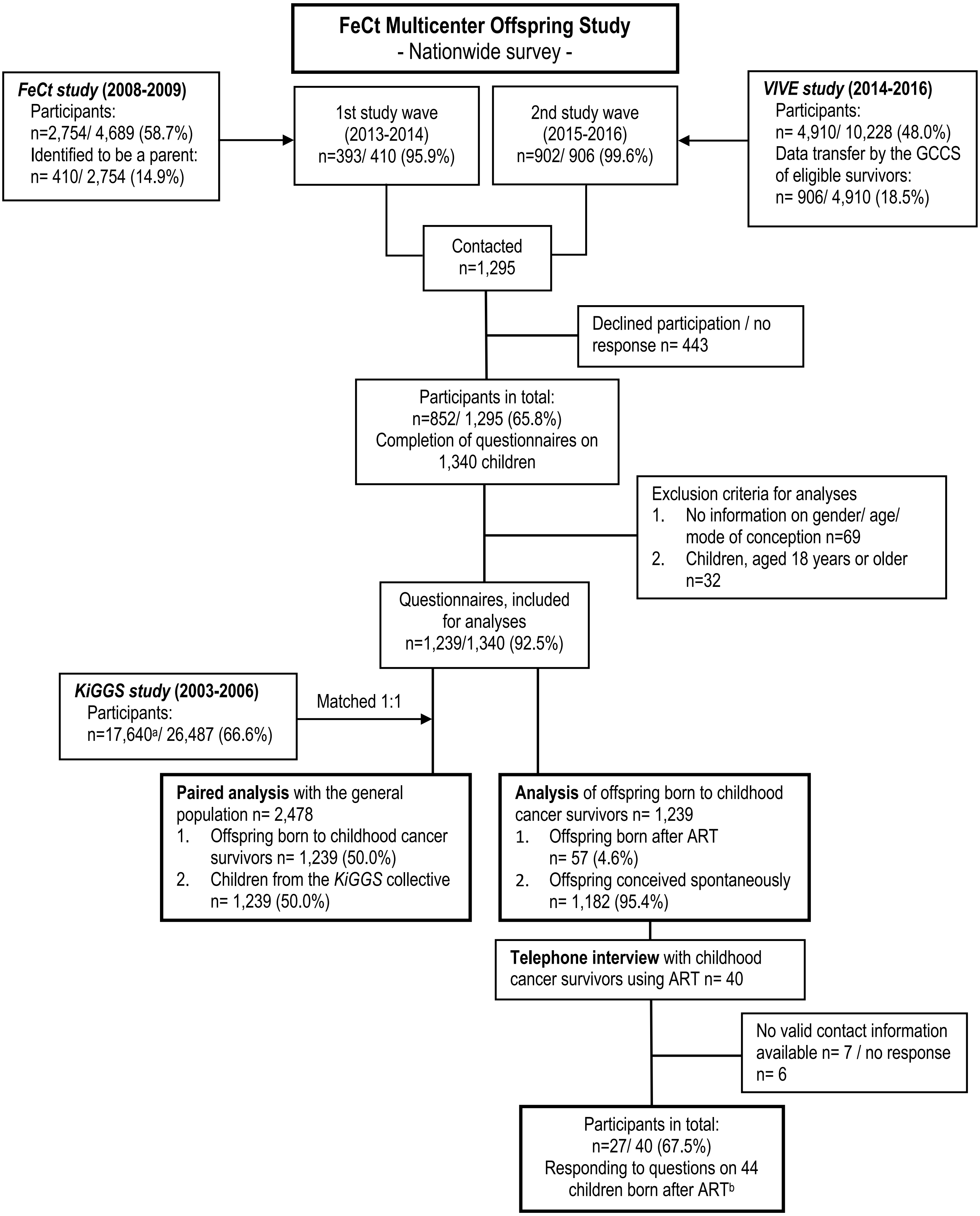

[a]retrospectively, data deletion was requested by one *KiGGS* participant
[b]including five children born after study participation

Acronyms & Abbreviations:

FeCt, Fertility after chemo- and radiation therapy in childhood and adolescence

GCCS, German Childhood Cancer Registry

ART, Assisted reproductive technology

Supplemental Table 1. Responder analysis of the contacted German childhood cancer survivors (n=1,295)

Characteristic	Responder		Non-Responder		*P*
	Missing Data (n)	n (%)	Missing Data (n)	n (%)	
**Total**		852 (100.0)		443 (100.0)	
**Gender**	-		-		<.001
Female		582 (68.3)		247 (55.8)	
Male		270 (31.7)		196 (44.2)	
**Age at time of survey**	-		-		.171
Mean age [SD]		33.4 [4.9]		33.8 [5.5]	
Median age [IQR]		33 [7.0]		33 [6.0]	
18 to 24		18 (2.1)		20 (4.5)	
25 to 29		176 (20.7)		74 (16.7)	
30 to 34		325 (38.1)		151 (34.1)	
≥ 35		333 (39.1)		198 (44.7)	
**Age at time of diagnosis**	-		-		.253
Mean age [SD]		8.4 [4.3]		8,7 [4.2]	
Median age [IQR]		9 [7.0]		10 [7.0]	
0 to 4		210 (24.6)		102 (23.0)	
5 to 9		238 (27.9)		111 (25.1)	
≥ 10		404 (47.4)		230 (51.9)	
**Diagnosis (ICCC-3)**	-		-		.199
Leukemia		361 (42.4)		191 (43.1)	
Lymphomas		124 (14.6)		83 (18.7)	
Brain tumors		63 (7.4)		36 (8.1)	
Neuroblastoma		29 (3.4)		9 (2.0)	
Retinoblastoma		17 (2.0)		10 (2.3)	
Renal tumors		60 (7.0)		29 (6.5)	
Hepatic tumors		3 (0.4)		1 (0.2)	
Bone tumors		78 (9.2)		33 (7.4)	
Soft tissue tumors		77 (9.0)		27 (6.1)	
Germ cell tumors		32 (3.8)		14 (3.2)	
Other malignant epithelial neoplasm		8 (0.9)		9 (2.0)	
Other neoplasm, unspecified		-		1 (0.2)	
**Educational level**	18		7		<.001
High		373 (44.7)		134 (30.7)	
Medium		339 (40.7)		164 (37.6)	
Low		122 (14.6)		138 (31.7)	

SD, standard deviation; IQR, interquartile range; ICCC-3, International Classification of Childhood Cancer, third revision (https://seer.cancer.gov/iccc/)

Supplemental Table 2. Multivariate analyses in offspring born to childhood cancer survivors[a]

Characteristic	Preterm birth		Low birth weight		Small for gestational age		Congenital malformations		Congenital heart defects	
	OR (95% CI)	*P*	OR (95% CI)	*P*	OR (95% CI)	*P*	OR (95% CI)	*P*	OR (95% CI)	*P*
**ART**	1.121 (0.449 to 2.797)	.806	2.395 (0.795 to 7.217)	.121	2.097 (0.859 to 5.116)	.104	0.47 (0.059 to 3.736)	.476	5.705 (0.155 to 209.576)	.344
**Female gender**	1.261 (0.844 to 1.884)	.257	0.881 (0.494 to 1.570)	.667	0.695 (0.436 to 1.109)	.127	0.694 (0.376 to 1.282)	.244	4.463 (1.180 to 16.878)	.028
**Age at time of survey**										
0 to 6	*Reference*	-	*Reference*	-	*Reference*	-	*Reference*	-	*Reference*	-
7 to 13	1.527 (0.969 to 2.408)	.068	1.785 (0.944 to 3.377)	.075	0.774 (0.435 to 1.375)	.381	2.281 (1.206 to 4.312)	.011	0.319 (0.081 to 1.260)	.103
≥ 14	2.485 (1.222 to 5.051)	.012	1.794 (0.623 to 5.163)	.279	2.179 (0.980 to 4.845)	.056	1.414 (0.393 to 5.084)	.596	0.039 (0.001 to 2.360)	.121
**Multiple-sibling birth**	3.902 (1.804 to 8.441)	.001	1.604 (0.508 to 5.063)	.420	1.894 (0.721 to 4.972)	.195	1.836 (0.398 to 8.478)	.436	0.596 (0.053 to 6.700)	.675
**Smoking during pregnancy**	2.157 (1.139 to 4.085)	.018	0.490 (0.164 to 1.468)	.203	1.519 (0.666 to 3.464)	.320	0.598 (0.172 to 2.070)	.417	1.952 (0.186 to 20.447)	.577
**Drinking during pregnancy**	1.014 (0.366 to 2.810)	.978	1.203 (0.265 to 5.454)	.811	0.890 (0.253 to 3.138)	.857	[b]	[b]	[b]	[b]
**Migration background**	1.281 (0.800 to 2.051)	.302	1.538 (0.791 to 2.991)	.204	1.567 (0.932 to 2.636)	.091	0.862 (0.401 to 1.851)	.703	0.463 (0.079 to 2.710)	.393
**Educational attainment**										
Low	*Reference*	-	*Reference*	-	*Reference*	-				
Medium	1.930 (0.953 to 3.910)	.068	1.894 (0.669 to 5.359)	.229	0.887 (0.435 to 1.808)	.741				
High	1.924 (0.926 to 3.999)	.080	1.506 (0.509 to 4.456)	.459	1.069 (0.514 to 2.223)	.858				
**Subjective health**										
Medium / poor / very poor	*Reference*	-	*Reference*	-	*Reference*	-	*Reference*	-	*Reference*	-
Good	0.581 (0.242 to 1.394)	.224	0.883 (0.238 to 3.271)	.852	0.710 (0.244 to 2.068)	.531	0.384 (0.132 to 1.121)	.080	2.111 (0.291 to 15.325)	.460
Very good	0.540 (0.228 to 1.277)	.161	1.103 (0.302 to 4.033)	.882	0.775 (0.274 to 2.194)	.631	0.183 (0.062 to 0.537)	.002	0.568 (0.082 to 3.950)	.568
**Preterm birth (<37 weeks)**			38.306 (21.044 to 69.727)	<.001						
**Congenital malformations**	2.985 (1.415 to 6.298)	.004	1.406 (0.431 to 4.581)	.572	2.568 (1.062 to 6.210)	.036			[b]	[b]
**Congenital heart defects**	1.142 (0.343 to 3.799)	.829	5.616 (1.030 to 30.610)	.046	0.514 (0.093 to 2.832)	.445	[b]	[b]		
**Age at time of diagnosis (survivor parent)**										
0 to 4	*Reference*	-	*Reference*	-	*Reference*	-	*Reference*	-	*Reference*	-
5 to 9	0.787 (0.441 to 1.402)	.416	1.758 (0.741 to 4.170)	.200	2.333 (1.184 to 4.597)	.014	1.012 (0.456 to 2.247)	.977	0.303 (0.061 to 1.500)	.143
≥ 10	0.908 (0.548 to 1.504)	.707	1.808 (0.826 to 3.959)	.138	1.346 (0.700 to 2.588)	.373	0.595 (0.274 to 1.289)	.188	0.362 (0.068 to 1.929)	.234
**Diagnosis (survivor parent,** grouped)										
Leukemia / Lymphomas	*Reference*	-	*Reference*	-	*Reference*	-	*Reference*	-	*Reference*	-
Brain tumors	0.735 (0.296 to 1.825)	.507	2.393 (0.870 to 6.580)	.091	2.256 (1.043 to 4.882)	.039	0.623 (0.141 to 2.750)	.533	14.634 (0.705 to 303.691)	.083
Extracranial solid tumors	1.240 (0.810 to 1.897)	.322	1.289 (0.697 to 2.386)	.419	1.423 (0.862 to 2.350)	.168	0.884 (0.455 to 1.717)	.716	1.28 (0.342 to 4.793)	.714

OR, odds ratio; CI, confidence interval; ART, assisted reproductive technologies
[a]Healthoutcome childhood cancers could not be taken into account for multivariate analyses as numbers were too small to report meaningful ORs.
[b]Numbers (n) were too small to report meaningful ORs

Supplemental Table 3. Multivariate analyses in offspring born to survivors and in the general population (*KiGGS study*)[a]

Characteristic	Preterm birth		Low birth weight		Small for gestational age		Congenital malformations		Congenital heart defects	
	OR (95% CI)	*P*	OR (95% CI)	*P*	OR (95% CI)	*P*	OR (95% CI)	*P*	OR (95% CI)	*P*
**Offspring born to survivors**	2.002 (1.395 to 2.874)	<.001	1.142 (0.704 to 1.854)	.590	1.227 (0.848 to 1.777)	.278	0.538 (0.364 to 0.796)	.002	1.156 (0.584 to 2.288)	.677
**Female gender**	1.178 (0.85 to 1.632)	.325	0.811 (0.519 to 1.266)	.356	0.654 (0.461 to 0.927)	.017	0.899 (0.623 to 1.298)	.571	1.528 (0.784 to 2.977)	.213
**Age at time of survey**										
0 to 6	*Reference*	-	*Reference*	-	*Reference*	-	*Reference*	-	*Reference*	-
7 to 13	1.422 (0.976 to 2.073)	.067	1.186 (0.710 to 1.982)	.515	0.821 (0.539 to 1.251)	.359	1.774 (1.206 to 2.609)	.004	0.547 (0.258 to 1.159)	.115
≥ 14	2.341 (1.304 to 4.206)	.004	1.053 (0.435 to 2.545)	.909	1.421 (0.719 to 2.808)	.312	0.489 (0.150 to 1.599)	.237	4.146 (0.951 to 18.087)	.058
**Multiple-sibling birth**	6.965 (4.156 to 11.674)	<.001	4.178 (2.033 to 8.589)	<.001	2.435 (1.267 to 4.680)	.008	1.917 (0.845 to 4.351)	.120	1.654 (0.504 to 5.436)	.407
**Smoking during pregnancy**	1.518 (0.923 to 2.498)	.100	0.773 (0.367 to 1.631)	.500	1.779 (1.098 to 2.882)	.019	0.753 (0.420 to 1.353)	.343	0.869 (0.288 to 2.623)	.804
**Drinking during pregnancy**	0.680 (0.338 to 1.367)	.279	0.907 (0.364 to 2.261)	.834	1.438 (0.811 to 2.547)	.214	0.871 (0.462 to 1.644)	.671	0.804 (0.250 to 2.592)	.715
**Migration background**	1.242 (0.844 to 1.829)	.271	1.431 (0.851 to 2.408)	.177	1.612 (1.093 to 2.377)	.016	0.919 (0.588 to 1.435)	.710	0.725 (0.308 to 1.705)	.461
**Educational attainment**										
Low	*Reference*	-	*Reference*	-	*Reference*	-				
Medium	1.682 (0.959 to 2.948)	.070	1.057 (0.508 to 2.202)	.882	0.838 (0.506 to 1.388)	.493				
High	1.643 (0.913 to 2.958)	.098	0.952 (0.437 to 2.073)	.901	0.861 (0.501 to 1.478)	.586				
**Subjective health**										
Medium / poor / very poor	*Reference*	-	*Reference*	-	*Reference*	-	*Reference*	-	*Reference*	-
Good	0.347 (0.196 to 0.615)	<.001	2.125 (0.786 to 5.746)	.138	0.999 (0.457 to 2.183)	.998	0.604 (0.323 to 1.127)	.113	1.049 (0.354 to 3.105)	.931
Very good	0.304 (0.171 to 0.540)	<.001	2.229 (0.818 to 6.071)	.117	1.062 (0.487 to 2.319)	.879	0.312 (0.161 to 0.604)	.001	1.088 (0.347 to 3.414)	.885
**Preterm birth**			35.217 (22.249 to 55.741)	<.001						
**Congenital malformations**	1.625 (0.888 to 2.975)	.116	1.318 (0.550 to 3.160)	.535	1.858 (1.000 to 3.450)	.050			[b]	[b]
**Congenital heart defects**	1.795 (0.726 to 4.436)	.205	2.510 (0.712 to 8.849)	.152	0.559 (0.171 to 1.832)	.337	[b]	[b]		

OR, odds ratio; CI, confidence interval

[a] Health outcome *childhood cancer* could not be taken into account for multivariate analyses as numbers were too small to report meaningful odds ratios
[b] Numbers were too small to report meaningful odds ratios
